# Structure-Based Discovery and Synthesis of Potential Transketolase Inhibitors

**DOI:** 10.3390/molecules23092116

**Published:** 2018-08-23

**Authors:** Jingqian Huo, Bin Zhao, Zhe Zhang, Jihong Xing, Jinlin Zhang, Jingao Dong, Zhijin Fan

**Affiliations:** 1 College of Plant Protection, Agricultural University of Hebei, Baoding 071001, China; huojingqian@163.com (J.H.); zhang_z2013@163.com (Z.Z.); 2 State Key Laboratory of Elemento-Organic Chemistry, College of Chemistry, Nankai University, No. 94, Weijin Road, Nankai District, Tianjin 300071, China; bdzhaobin@126.com; 3 College of Life Science, Agricultural University of Hebei, Baoding 071000, China; xingjihong2000@126.com; 4 Collaborative Innovation Center of Chemical Science and Engineering (Tianjin), Nankai University, Tianjin 300071, China

**Keywords:** transketolase, virtual screening, amides derivatives, synthesis, biological activity

## Abstract

Transketolase (TKL) plays a key role in plant photosynthesis and has been predicted to be a potent herbicide target. Homology modeling and molecular dynamics simulation were used to construct a target protein model. A target-based virtual screening was developed to discover novel potential transketolase inhibitors. Based on the receptor transketolase 1 and a target-based virtual screening combined with structural similarity, six new compounds were selected from the ZINC database. Among the structural leads, a new compound ZINC12007063 was identified as a novel inhibitor of weeds. Two novel series of carboxylic amide derivatives were synthesized, and their structures were rationally identified by NMR and HRMS. Biological evaluation of the herbicidal and antifungal activities indicated that the compounds **4u** and **8h** were the most potent herbicidal agents, and they also showed potent fungicidal activity with a relatively broad-spectrum. ZINC12007063 was identified as a lead compound of potential transketolase inhibitors, **4u** and **8h** which has the herbicidal and antifungal activities were synthesized based on ZINC12007063. This study lays a foundation for the discovery of new pesticides.

## 1. Introduction

Weed control in agricultural crops is a global problem. Chemical control has become the best choice for weed control since the emergence of 2,4-dichlorophenoxyacetic acid (2,4-D) in the 1940s. However, with the large number and long-term use of chemical herbicides, the negative effects of weed resistance and ecological pollution have gradually been revealed [1,2]. Traditional pesticide development not only requires a long period of time but also more financial resources. Accordingly, it is urgently necessary to develop green highly efficient and environmentally friendly green herbicides. With the advances in molecular biology and the widespread application of computer technology, the combination of computer simulation, chemical synthesis and biological testing represents has been became a new strategy for drug development. Currently, computer simulation is widely used in the field of medicine, but it is rarely used in the field of herbicide development.

The enzyme transketolase is a limiting factor of photosynthesis, and small changes in its activity can lead to the inhibition of plant growth as well as phenylalanine and aromatic amino acid metabolism [3,4,5,6]. Since transketolase plays a key role in plant photosynthesis, and is highly conserved in plants, it represents an important candidate target for the development of new herbicides. Henkes [3] found that transketolase can be used as a target for new herbicide development, but inhibitors for this enzyme have not been reported up until now.

Virtual screening is a cost-effective and efficient strategy in drug discovery. It involves searching small molecule libraries to identify those structures that are most likely to bind to a biological target, which is typically a proteinreceptor or enzyme [7]. Molecular docking is a drug screening method based on the target protein three-dimensional (3D) structure. Through the molecular docking of small molecules to the target, the spatial conformation can be analyzed, including the nature of the electrostatic interactions, hydrogen bond, hydrophobic interactions and van der Waals forces [8]. Accordingly, virtual screening is a good tool for new compound discovery. In molecular docking, to perform pharmacophore screening or shape similarity screening, a database with a larger diversity of small molecules should be selected. For this purpose, we chose ZINC free database which contains fragment libraries, drug pools, drug libraries, natural product libraries, etc. fragment libraries, drug pools, drug libraries, natural product libraries, etc. [9].

In this study, the ZINC free database was screened by virtual screening using transketolase as the target to obtain the potential transketolase inhibitors, and two novel series of amide derivatives were designed and synthesized based on the structural leads, which could increase the structural diversity and discover more potent inhibitors of transketolase.

## 2. Results and Discussion

### 2.1. Homology Modeling of AtTKL1

**Figure 1:**
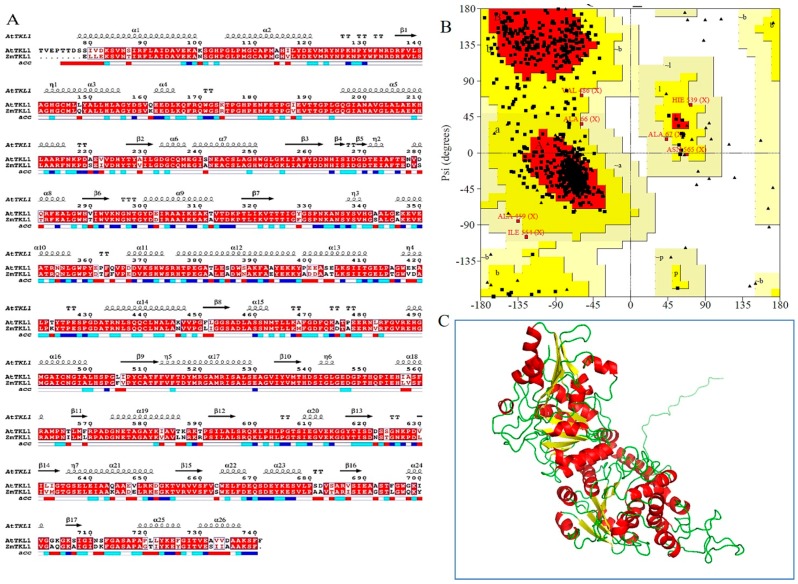
The bioinformatics analysis of transketolase. (**A**) Structure alignment between AtTKL1 in *A. thaliana* and ZmTKL1 in *Zea mays*; (**B**) Ramachandran Plot of the protein after structural refinement; (**C**) Refined structure of the AtTKL1 protein, where helix is shown in red, yellow indicates beta sheets, and coil is shown in green color.

The structure of *Arabidopsis thaliana* transketolase (AtTKL1) was established by Modeller. The sequence of AtTKL1 (ID: AT3G60750) was obtained from the TAIR database, and *Zea mays* (corn) transketolase ZmTKL1 (PDB, 1ITZ_A) [10] was chosen as the homologous model. The homology was 85%, and the sequence coverage was 90%. The sequence alignment for modeling and the structural elements are shown in Figure 1A, the structures of ZmTKL1 and AtTKL1 have similar tertiary structure. The features of an α helix, β sheet and r coil are conserved in both protein structures. The model of AtTKL1 was optimized by dynamic simulation with Amber12 after 500 ps, and the stability of the architecture was verified again. The final model was obtained by MD simulations to achieve the stable 3D structure of AtTKL1 (Figure 1C). The stability of the architecture was verified again. The residues in most of the favored regions [A, B, L] were 80.7%; the residues in the additional allowed regions [a, b, l, p] were 18.2%; the residues in the generously allowed regions [~a, ~b, ~l, ~p] were 1.1%; and the residues in the disallowed regions were 0% (Figure 1B). All of the parameters were suitable for virtual screening.

### 2.2. Virtual Screening and Biological Activity

Transketolase is a target for herbicide design [3,11], and it has a highly conserved homologous domain in higher plants and fungi. A total of 25 candidate molecules with binding affinities lower than −8.5 kcal/mol (Table S1) were obtained from 1 million ZINC compounds after virtual screening. Some compounds had similar structures, and thus we selected six small molecules that had a lower affinity and different structures (Table 1). The combination of a small molecule model and transketolase was simulated using PyMOL [12,13], and the details of the docking study of AtTKL1 with ligand molecules are shown in Table 1. These small molecules could be bound by hydrogen bonding. These compounds had similar molecular weights (350–500), XlogP (1.5–4.0), hydrogen donor (1–3), hydrogen acceptor (6–10) and H-bond binding sites (Table 1, Figure S1). All of the selected compounds were purchased from the ZINC database vendor. The biological activities of those compounds were tested against rape and barnyard grass with a dosage of 500 mg/L. The bioassay indicated that ZINC12007063 displayed moderate to good herbicidal activity, and it had a similar herbicidal activity to the commercial herbicide atrazine (Table 2).

With the development of computer technology and the emergence of the post genome era, there is a new way to discover new drugs by computer-based virtual screening technology, which can provide an opportunity for the re-identification of active compounds. In recent years, researchers have used molecular docking technology to find an increasing number of lead compounds. Docking calculations have been applied in pharmaceutical research for nearly two decades. To date, the researchers have identified two million active compounds from approximately 500 targets. Admittedly, this technology is still mainly used in the field of medicine, and it has not been widely used in the development of pesticides.

In agriculture, Yuan et al. [14] has screened antifungal drugs from the ZINC database with CYP51, which is an essential enzyme in sterol biosynthesis. A novel herbicide of urease inhibitors has also been identified by virtual screening and in vitro assays [15]. However, with the advances of science and technology, more of these new technologies will be applied to the field of pesticide development.

### 2.3. The Syntheses and Biological Activities of the New Compounds

**Scheme 1:**
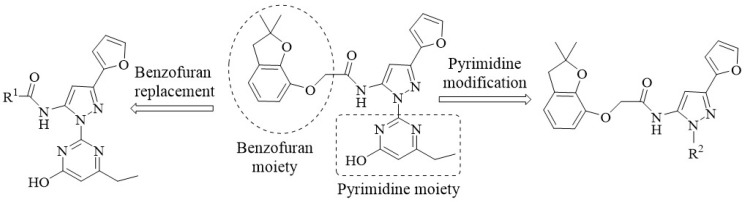
The design strategy for new compounds.

**Scheme 2:**
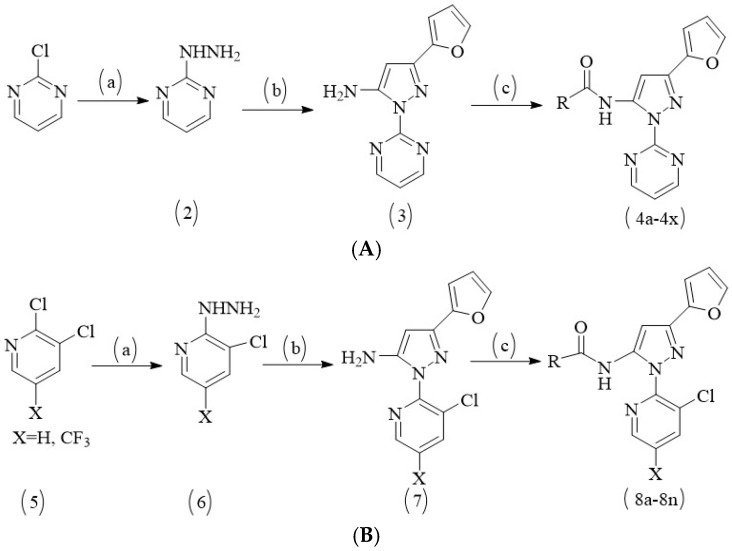
Synthetic route for compounds **4a**–**4x** and **8a**–**8n**. (**A**) *Reagents and conditions for* **4a**–**4x**: (a) absolute ethanol, hydrazine hydrate, refluxing for 90 min; (b) absolute ethanol, 2-furoylacetonitrile, refluxing for 120 min; (c) anhydrous DCM, substituted acyl chloride, Et_3_N, room temperature, overnight. (**B**) *Reagents and conditions for* **8a**–**8n**: (a) absolute ethanol, hydrazine hydrate, refluxing for 180 min; (b) absolute ethanol, 2-furoylacetonitrile, refluxing for 180 min; (c) anhydrous DCM, substituted acyl chloride, Et_3_N, room temperature, overnight.

Virtual screening and biological activity analysis showed that ZINC12007063 had a good herbicidal activity. Basing on compound ZINC12007063, two novel series of carboxylic amides were designed (Scheme 1) and synthesized. The two-novel series of carboxylic amides (**4a**–**4x**) and (**8a**–**8n**) were synthesized as described in Scheme 2, and the structures are shown in Table 2. 2-Furoylacetonitrile, which is required in the synthesis of both series of compounds **4a**–**4x** and **8a**–**8n**, was prepared from methyl-2-furoate. The reaction of methyl-2-furoate with CH_3_CN in the presence of NaH in toluene at the refluxing temperature for 24 h gave 2-furoylacetonitrile. 2-Furoylacetonitrile was then reacted with 2-hydrazinepyrimidine or 2,3-dichloro-5-substituted pyridine in absolute ethanol at the refluxing temperature to afford 3-(furan-2-yl)-1-pyrimidin-1*H*-pyrazol-5-amine (**3**) or 3-(furan-2-yl)-1-(3-dichloro-5-substituted pyridine-2-yl)-1*H*-pyrazol-5-amines (**7**). The substituted acyl chlorides were obtained by the reaction of the corresponding carboxylic acids with SOCl_2_ and a catalytic amount of DMF at the refluxing temperature. Compounds **4a**–**4x** and **8a**–**8n** were synthesized by the condensation reaction of the substituted acyl chlorides with intermediates **3** or **7** using Et_3_N as the base and CH_2_Cl_2_ as the solvent. 

The reaction temperature was very important for the cyclization reaction of 2-furoylacetonitrile with 3-(furan-2-yl)-1-pyrimidin-*H*-pyrazol-5-amine (**3**) or 3-(furan-2-yl)-1-(3-dichloro-5-substituted pyridine-2-yl)-*H*-pyrazol-5-amines (**7**) and a by-product was detected unless the reaction was performed at reflux temperature. The crude products were purified by column chromatography over silica gel (200–300 mesh) using ethyl acetate-petroleum ether or dichloromethane-methanol as the eluent to afford pure products **4a**–**4x** and **8a**–**8n**.

The chemical structures of the newly synthesized compounds were established on the basis of analytical and spectroscopic data. In the ^1^H-NMR spectra, the chemical shifts for the amide NH were found at δ = 13.74–10.48 ppm as singlets. The aromatic proton signals appeared at δ = 9.24–6.43 ppm. The signals for OCH_3_ and CH_2_ were observed at δ = 3.91–3.89 and 4.82–3.09 ppm, respectively. Furthermore, all compounds were confirmed by ESI-MS and were in agreement with their molecular weight. The molecular structure of compound **4b** is shown in Figure S2. X-ray single-crystal diffraction analysis further confirmed the structure of compound **4b**. The appearance, yields, melting points and spectral data of the synthesized compounds are described in the Supporting Information.

### 2.4. The Biological Activities of the New Compounds ***4a**–**4x*** and ***8a**–**8n***

*A. thaliana* is a widely used model plant which could be used to develop a homology model, and *Brassica campestris* L. (rape) and *Echinochloca crus-galli* L. (barnyard grass) were the two representative plants used to measure the herbicidal activity. All of the synthesized compounds **4a**–**4x**, **8a**–**8n** were evaluated for herbicidal activity against rape and barnyard grass at dosages of 500 mg/L. Some compounds displayed moderate to good herbicidal activity against rape and barnyard grass in the bioassays (Table 2). For example, **4u** exhibited inhibitory rates of >80% towards the root growth of barnyard grass, and **8h** showed inhibitory rates of >80% towards the root growth of rape. However, some compounds showed poor or no herbicidal activity against rape and/or barnyard grass. In general, the herbicidal activities of compounds **8a**–**8n** were higher than those of compounds **4a**–**4x**, which indicated that the introduction of a chloro or trifluoromethyl substituent could improve the herbicidal activity. For example, compound **8h** displayed higher herbicidal activities than compound **4t**, which had no chloro or trifluoromethyl substituent. In addition, **4n**, **4q**, **4r**, **4s** and **8d** showed stronger inhibition against the growth of the dicotyledon rape than that of the monocotyledon barnyard grass, and **4e** as well as **4w** showed stronger inhibition against the growth of the barnyard grass than that of the rape. Furthermore, these compounds exhibited a relative selectivity.

Transketolase is widely distributed in microorganisms, such as fungi, bacteria, yeast and so on. Therefore, the inhibitory effects of all of the synthesized compounds **4a**–**4x**, **8a**–**8n** against six typical fungi were evaluated at dosages of 50 mg/L in vitro. Azoxystrobin was used as a positive control. Some compounds showed good fungicide activity against the selected fungi (Table 2). All of the compounds showed moderate to good antifungal activity against the strain of *S. sclerotioru*. Compound **4x** presented the best activity against the strain of *C. arachidicola*, with an inhibition rate of 64.56%, which was higher than that of azoxystrobin. The inhibition rates of compounds **4f**, **4g** and **4n** against *P. sasakii* were above 50%. Compounds **4f**, **4g**, **4n**, **8a**, **8c** and **8e** had relatively broad-spectrum fungicidal activity.

Bio-rational design of new pesticide molecules based on target genes or proteins plays an important role in the current new pesticide creation process and is an important way to develop new pesticides [16,17,18]. Therefore, the search and verification of new drug targets will become hot in the development of new drugs [19,20,21]. In addition, design and development of novel eco-friendly pesticides based on a new target [22] is of great importance to solve the resistance problem. Optimizing the structure of a lead compound, which is obtained based on the new target, will give a chance to develop more potent inhibitors. In this study, we identified compound ZINC12007063 with good herbicidal activity according to the virtual screening based on transketolase. To obtain more potential transketolase inhibitors, some heterocyclic groups were introduced to modify the lead compound structure according to the bio-electronic isotype principle and at last two novel series of carboxylic amide derivatives were synthesized. The bioassay results of the synthesized compounds indicated that the herbicidal activities and fungicide activities of compounds **4u** and **8h** were all better than those of the lead compound ZINC12007063. The design and synthesis of compounds with multiple activities at the same time seems difficult, and currently, there are few reports in this area. Zhang et al. have synthesized 5-substituted-1,3,4-oxadiazole Mannich bases and bis-Mannich bases with a target ketol-acid reductoisomerase, which exhibited obvious herbicidal activity against *Echinochloa crus-galli* and fungicidal activities against *Rhizoctonia cerealis* and *Physalospora piricola* [23,24]; Zhao et al. have designed and synthesized a series of novel triazines containing arylmethyl amino moieties, but some compounds only have good activity on plants or fungi [25].

### 2.5. Molecular Docking of Compounds ***4u*** and ***8h*** with Transketolase

**Figure 2:**
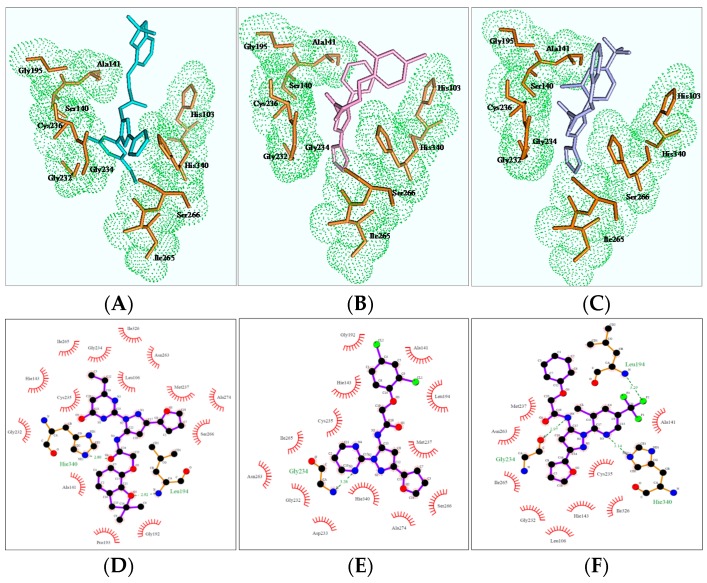
Binding mode of ZINC12007063 (**A**,**D**), **4u** (**B**,**E**) and **8h** (**C**,**F**).

In order to better understand the mechanism of newly designed potential transketolase inhibitors, all the derivatives were docked into the active site of AtTKL1. The detailed interactions of compounds ZINC12007063, **4u** and **8h** with AtTKL1 are showed in Figure 2. In the docked complex of compound ZINC12007063 and AtTKL1 (Figure 2A,D), the benzene ring benzotetrahydrofuran of ZINC12007063 interacted with His103 through a π-π interaction. Moreover, the tetrahydrofuran ring and O^4^ of ZINC12007063 formed a hydrogen bond with catalytic site residues Leu194 and His340 respectively, which were importantly catalytic site in AtTKL1^20^. Through docking analysis between **4u** with AtTKL1 (Figure 2B,E) and **8h** with AtTKL1 (Figure 2C,F), we found a similar combination pattern. In addition, the compounds **4u** and **8h** can combined Gly234 of AtTKL1 with hydrophobic interaction. All of these favorable interactions may result in the potent bioactivity of **4u** and **8h**.

Thus, the changes in some functional groups did not affect ligand-protein binding, but biological activity was better than the control. In the future, we will design new compounds based on the quantitative structure-activity relationship analysis and consider the affinity of the designed compounds with transketolase based on molecular docking. Therefore, this study will lay the foundation for the further design and synthesis of new bioactive compounds.

## 3. Materials and Methods

### 3.1. Homology Modeling of A. thaliana Transketolase 1 (AtTKL1)

The protein sequence of *A. thaliana* transketolase 1 (AtTKL1) was obtained from the TAIR database. The Modeller 9.13 software is used for the modeling of protein 3D structures [26,27,28]. Molecular dynamics and energy minimization were carried out using Amber12 according to standard approaches [29]. The structural evaluation for AtTKL1 was performed by the ERRAT server [30], Procheck server [31], and Verify3d server [32].

### 3.2. Virtual Screening

The almost one million chemical ligand structures used in this study were downloaded from the ZINC database (http://zinc.docking.org). The PDBQT files of the receptor and ligands were converted by Open Babel 2.3.2 [33]. Then, the receptor and ligand docking studies were conducted using the AutoDock Vina program [34].

### 3.3. Biological Activities of Compounds

#### 3.3.1. Herbicidal Bioassay

The effects of the compounds on inhibiting the growth of dicotyledonous rape (*B. campestris* L.) and monocotyledonous barnyard grass (*E. crus-galli* L.) were assessed using the small cup method. Generally, the compound (2 mg) was dissolved in acetone (80 μL). Then, the solution was diluted with water containing 0.2% Tween 80 to reach a concentration of 500 mg/L. The mixture of the same amount of water, acetone, and Tween 80 was used as a control. Barnyard grass seeds and rape seeds were soaked in distilled water for 12 h before being placed on a filter paper in a 5 cm cup, to which 1 mL of inhibitor solution had been added in advance. Usually, 10 barnyard grass seeds and 10 rape seeds were used for each cup. Each treatment was repeated three times. The cup was placed in an illumination incubator and kept at 25 °C, with light of 10 µmol/m^2^/s for 12 h each day. After incubation for 7 days, the root and stalk of two test plants were measured in each cup, respectively. The percentage inhibition, which is based on the measurement of root length (or stalk length) compared to an untreated control, was used to describe the control efficiency of the compound.

#### 3.3.2. Fungicide Bioassay

The effects of the compounds on inhibiting the growth of fungi were assessed using the fungi growth inhibition method with potato dextrose agar (PDA) as the cultivation medium. Generally, 2 mg of the compound was dissolved in 200 μL of *N*,*N*-dimethylformamide (DMF), and then, 3.8 mL of sterilized water was added to obtain a stock solution with a concentration of 500 mg/L. One milliliter of the stock solution was transferred into a 10 cm diameter Petri dish. Plates were prepared with 9 mL of PDA. Before the plate solidification, the PDA was thoroughly mixed by turning the Petri dish in the sterilized hood 5 times to scatter the compound evenly in the PDA. Next, each plate was inoculated with a 4 mm diameter fungi cake and cultured in the culture tank at 24~26 °C. Each treatment was repeated three times. The diameter of the fungal spread was measured 2 days later. The growth inhibition was then calculated using the corresponding blank.

### 3.4. Designing and Synthesizing New Compounds as Potent Herbicide Candidates

To increase the structural diversity and discover more potent inhibitors of transketolase, it is important to design new compounds based on the structures of candidate compounds that have good activities. All reagents and solvents for the syntheses and analyses were of analytical grade and used without further treatment unless otherwise stated. Column chromatography purification was carried out by using silica gel (200~300 mesh). The melting points were measured on an XT-4A apparatus, and are uncorrected. NMR spectra were obtained on an AV-400 spectrometer (Bruker, Rheinstetten, Germany) operating at 400 MHz for ^1^H NMR and 100 MHz for ^13^C NMR using CDCl_3_ or DMSO-*d*_6_ as solvents and TMS as the internal standard. The high-resolution mass spectra were recorded on a 6520-QTOF LC/MS (Agilent, Santa Clara, CA, USA) with an ESI source in the positive ion mode. The single-crystal structure was determined on a Saturn 724 CCD diffractometer (Rigaku, Woodlands, TX, USA).

## 4. Conclusions

Transketolase is a target for herbicide design, and a highly active lead compound ZINC12007063 was identified by virtual screening. Based on this lead compound, compounds **4u** and **8h**, two carboxylic amide derivatives, were synthesized, and both have herbicidal and antifungal activities. This study will lay a foundation for discovering new pesticides.

## Figures and Tables

**Table 1:** The structure of inhibitors which obtained by virtual screening.

No.	ZINC ID and Chemical Name	Affinity (kcal/mol)	xlogP	Molecular Weight (g/mol)	H-Bond Donors/Acceptors	H-Bond Binding Site/Bond Distance	Structural Formula
a	ZINC12007063 2-[(2,2-dimethyl-3*H*-benzofuran-7-yl)oxy]*-N*-[2-(4-ethyl-6-oxo-1H-pyrimidin-2-yl)-5-(2-furyl)pyrazol-3	−9.1	3.81	474.497	1/10	Ser266/3.3Å	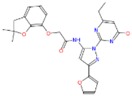
						Ser140/3.0Å	
						Gly232/3.4Å	
						His340/3.3Å	
						Leu194/2.9Å	
						Ser266/2.9Å	
b	ZINC12126699 *N*-[5-cyclopropyl-2-(4-isopropyl-6-oxo-1*H*-pyrimidin-2-yl)pyrazol-3-yl]-2-[(2,2-dimethyl-3*H*-benzofuran	−9.0	4.07	462.53	1/9	Ser266/3.3Å	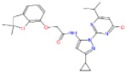
						Ser140/3.0Å	
						Gly232/3.4Å	
						His340/3.3Å	
						Leu194/2.9Å	
c	ZINC19961402 (5*R*,6*S*)-5(3-chlorophenyl)-4,7-dioxo-2-(phenylamino)-3,4,5,6.7,8-hexahydroquinazoline-6-carbonitrile	−9.0	1.61	391.818	3/7	His143/3.2Å	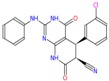
						Gly/234/3.5Å	
						Gly232/3.1Å	
d	ZINC16283531 (NE)-*N*-[[(4,6-dimethylpyrimidin-2-yl)amino]-[[3-(trifluoromethyl)phenyl]amino]methylene]-2,2-dimethyl	−8.7	4.33	392.405	1/6	Gly234/3.2Å	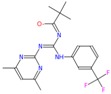
e	ZINC58191888 (4*Z*)-4-[[4-[(1-methylimidazol-2-yl)methoxy]phenyl]-methyslene]isoquinoline-1,3-dione	−8.6	2.5	359.385	1/6	His103/3.2Å	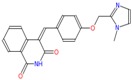
						His340/3.5Å	
						Cys235/3.1Å	
f	ZINC12929396 3-isopropyl-*N*-methyl-*N*-[(4-oxo-3H-quinazolin-2-yl)methyl]isoxazolo[4,5-e]pyridine-5-carboxamide	−8.6	3.46	377.404	1/8	Gly232/3.4Å	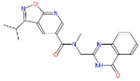
						His340/3.1Å	
						Gly192/3.5Å	

**Table 2:** Bioactivity of compounds (**4a**–**4x** and **8a**–**8n**) through growth inhibition method (inhibition rate %, herbicide activity at 500 mg/L and fungicide activity at 50 mg/L).

Compound	Substituent Group		Herbicidal Activity				Fungicide Activity					
	*R*	*X*	*Brassica campestris*		*Echinochloca crus-galli*		*C. arachidicola*	*B. cinerea*	*G. zeae*	*S. sclerotiorum*	*R. cerealis*	*P. sasakii*
			Root	Stem	Root	Stem						
**4a**	7-ethoxybenzofuran	/	0	0	0	0	46.24 ± 1.63	30.24 ± 2.94	19.18 ± 1.63	97.18 ± 2.16	0	39.65 ± 4.32
**4b**	pyridin-2-yl	/	0	0	4.15 ± 1.63	0	31.24 ± 1.63	50.95 ± 0.82	26.34 ± 3.56	85.35 ± 2.94	9.46 ± 1.41	43.96 ± 2.16
**4c**	pyridin-3-yl	/	31.30 ± 2.16	6.12 ± 1.63	14.24 ± 2.45	25.59 ± 2.45	8.57 ± 1.41	0	19.57 ± 1.63	96.09 ± 2.94	9.94 ± 3.74	30.28 ± 1.41
**4d**	pyridin-4-yl	/	43.12 ± 5.35	0	26.32 ± 2.94	10.37 ± 1.41	31.36 ± 2.16	21.28 ± 0.82	26.02 ± 0.82	98.12 ± 1.63	9.42 ± 7.12	0
**4e**	6-CF_3_-pyridin-2-yl	/	0	0	73.85 ± 9.27	36.22 ± 1.41	27.97 ± 1.63	60.43 ± 0.82	43.86 ± 2.16	81.39 ± 1.63	14.97 ± 2.16	28.66 ± 3.56
**4f**	thiophene-2-yl	/	0	0	0	0	46.52 ± 1.41	35.28 ± 1.63	37.56 ± 1.41	73.92 ± 3.56	39.38 ± 7.12	63.17 ± 1.63
**4g**	furan-2-yl	/	27.21 ± 3.74	0	25.24 ± 1.41	10.26 ± 2.16	42.30 ± 2.16	33.37 ± 0.82	65.26 ± 5.89	100	17.49 ± 2.83	52.28 ± 2.94
**4h**	Ph	/	0	0	23.32 ± 4.32	7.15 ± 2.94	31.61 ± 0.82	70.29 ± 0.82	30.15 ± 0.82	100	9.28 ± 4.32	0
**4i**	2-CF_3_-Ph	/	0	0	0	0	15.23 ± 2.83	50.49 ± 0.82	22.50 ± 8.29	96.01 ± 2.83	0	0
**4j**	3-CF_3_-Ph	/	0	0	22.18 ± 3.74	5.18 ± 2.16	23.06 ± 0.82	30.58 ± 2.94	15.35 ± 1.63	100	4.55 ± 1.41	43.09 ± 6.98
**4k**	4-CF_3_-Ph	/	17.02 ± 1.63	0	0	0	8.41 ± 0.82	50.09 ± 1.63	26.64 ± 1.41	100	9.37 ± 8.52	33.17 ± 2.45
**4l**	3-F-Ph	/	19.65 ± 2.94	23.11 ± 1.41	0	0	46.52 ± 2.94	55.98 ± 1.63	41.53 ± 2.16	96.94 ± 1.63	0	33.92 ± 10.2
**4m**	4-F-Ph	/	71.14 ± 1.63	45.20 ± 3.56	61.25 ± 2.45	35.38 ± 4.32	0	30.94 ± 2.94	4.29 ± 3.56	96.37 ± 2.83	9.25 ± 1.63	22.40 ± 3.27
**4n**	2-F-Ph	/	69.30 ± 2.94	22.45 ± 2.94	12.14 ± 2.94	0	31.61 ± 0.82	50.37 ± 0.82	59.58 ± 2.45	94.38 ± 3.74	9.34 ± 2.94	52.38 ± 5.89
**4o**	2-Cl-Ph	/	19.08 ± 3.27	8.18 ± 3.74	21.15 ± 1.63	5.15 ± 2.94	36.99 ± 2.16	40.29 ± 0.82	57.30 ± 5.35	93.28 ± 2.45	23.36 ± 2.16	24.19 ± 2.94
**4p**	3-Cl-Ph	/	0	0	0	0	27.16 ± 1.63	45.38 ± 2.16	43.91 ± 0.82	71.30 ± 0.82	14.63 ± 2.94	20.28 ± 0.82
**4q**	4-Cl-Ph	/	32.25 ± 3.60	57.14 ± 3.56	6.18 ± 2.16	0	27.12 ± 3.56	50.49 ± 4.08	48.64 ± 1.41	69.07 ± 4.97	18.28 ± 2.45	8.07 ± 2.16
**4r**	2,6-F_2_-Ph	/	59.66 ± 3.27	40.02 ± 2.16	0	0	36.35 ± 1.41	60.38 ± 0.82	24.28 ± 0.82	64.32 ± 1.41	18.91 ± 11.43	20.59 ± 2.94
**4s**	2,4-Cl_2_-Ph	/	60.29 ± 1.63	25.30 ± 2.16	2.92 ± 2.16	0	18.81 ± 0.82	65.94 ± 1.63	29.17 ± 1.63	79.59 ± 4.32	14.28 ± 5.72	24.94 ± 1.63
**4t**	PhOCH_2_CH_2_	/	54.48 ± 2.94	41.01 ± 2.45	33.45 ± 4.32	10.18 ± 4.32	55.49 ± 2.94	50.28 ± 2.16	48.61 ± 1.41	62.27 ± 5.72	36.97 ± 3.56	32.97 ± 0.82
**4u**	2,4-Cl_2_-PhOCH_2_CH_2_	/	66.70 ± 0.82	73.15 ± 2.83	83.52 ± 2.16	55.26 ± 2.45	27.61 ± 0.82	65.37 ± 0.82	62.28 ± 1.63	50.38 ± 2.94	36.29 ± 2.94	12.08 ± 2.94
**4v**	4-NO_2_-Ph	/	25 ± 2.45	0	0	0	36.35 ± 1.63	55.28 ± 1.63	43.09 ± 1.41	69.29 ± 2.45	14.52 ± 1.41	28.33 ± 3.74
**4w**	4-MeO-Ph	/	0	0	58.24 ± 2.45	33.23 ± 2.16	45.19 ± 1.63	75.68 ± 2.94	30 ± 2.45	79.21 ± 1.41	23.88 ± 2.16	12.28 ± 2.16
**4x**	cyclopropyl	/	0	11.04 ± 1.63	26.40 ± 4.24	10.12 ± 2.16	64.56 ± 2.94	70.64 ± 1.63	33 ± 0.82	79.28 ± 3.27	23.34 ± 3.74	32.17 ± 0.82
**8a**	3-CF_3_-Ph	H	53.15 ± 3.56	17.10 ± 2.16	9.20 ± 3.27	20.02 ± 6.48	47.89 ± 4.97	25.28 ± 4.32	37.17 ± 0.82	85.59 ± 3.56	24.09 ± 2.16	39.16 ± 4.08
**8b**	3-Cl-Ph	H	56.22 ± 1.63	41.24 ± 2.16	47.48 ± 1.63	16.65 ± 3.74	40.39 ± 0.82	58.46 ± 0.82	40.76 ± 1.41	98.03 ± 1.63	10.29 ± 2.94	43.24 ± 3.56
**8c**	PhOCH_2_CH_2_	H	73.27 ± 2.94	60.55 ± 1.41	38.17 ± 3.56	10.15 ± 1.63	53.19 ± 2.16	67.35 ± 1.41	43.16 ± 0.82	93.49 ± 2.16	19.87 ± 4.32	35.49 ± 1.63
**8d**	2,4-Cl_2_-PhOCH_2_CH_2_	H	76.25 ± 0.82	29.58 ± 2.16	21.18 ± 4.08	5.47 ± 4.32	27.18 ± 1.63	55.88 ± 1.63	40.25 ± 0.82	76.58 ± 2.16	5.24 ± 1.63	24.28 ± 1.41
**8e**	4-MeO-Ph	H	27.49 ± 2.16	13.22 ± 2.83	43.54 ± 5.89	31.24 ± 4.55	18.49 ± 2.83	50.92 ± 0.82	57.28 ± 6.53	76.19 ± 2.16	9.91 ± 1.41	16.57 ± 2.16
**8f**	6-CF_3_-pyridin-2-yl	H	31.54 ± 3.74	0	21.40 ± 1.41	5.18 ± 1.63	36.48 ± 3.74	55.38 ± 0.82	38.66 ± 3.74	71.49 ± 0.82	27.28 ± 3.74	36.39 ± 3.56
**8g**	3-CF_3_-Ph	CF_3_	52.22 ± 6.48	19.44 ± 4.24	15 ± 2.94	0	27.18 ± 1.63	54.19 ± 0.82	69.19 ± 1.63	93.38 ± 2.16	0	35.02 ± 0.82
**8h**	PhOCH_2_CH_2_	CF_3_	82.48 ± 7.26	72.25 ± 4.55	41.15 ± 1.63	11.08 ± 4.08	27.19 ± 1.41	25.28 ± 2.16	37.17 ± 2.94	95.06 ± 3.74	19.21 ± 2.83	26.89 ± 3.56
**8i**	3-Cl-Ph	CF_3_	48.47 ± 2.94	26.15 ± 2.16	8.18 ± 2.16	0	27.49 ± 3.27	50.94 ± 2.16	62.91 ± 8.83	74.15 ± 1.41	0	24.34 ± 2.83
**8j**	2-Cl-Ph	CF_3_	35.26 ± 4.32	40.25 ± 2.16	20.18 ± 2.16	1.08 ± 0.82	36.52 ± 1.63	20.35 ± 1.41	37.09 ± 2.45	74.27 ± 1.41	14.19 ± 1.63	20.28 ± 9.42
**8k**	4-Cl-Ph	CF_3_	18.30 ± 2.94	29.02 ± 2.45	3.24 ± 1.41	0	9.87 ± 1.41	50.97 ± 2.16	24.28 ± 0.82	83.59 ± 4.55	23.28 ± 1.41	24.19 ± 2.45
**8l**	4-NO_2_-Ph	CF_3_	2.19 ± 1.41	0	3.20 ± 2.16	9.20 ± 3.56	27.91 ± 3.27	65.97 ± 0.82	38.17 ± 3.56	57.14 ± 2.94	27.09 ± 4.55	20.28 ± 1.41
**8m**	4-MeO-Ph	CF_3_	12.18 ± 2.94	11.15 ± 3.27	44.18 ± 2.16	13.30 ± 2.45	45.49 ± 1.63	60.68 ± 1.63	62.29 ± 0.82	52.79 ± 7.35	14.68 ± 1.41	16.47 ± 2.16
**8n**	6-CF_3_-pyridin-2-yl	CF_3_	4.24 ± 2.94	2.99 ± 2.16	0	0	9.35 ± 2.16	50.94 ± 2.16	38.89 ± 4.55	67.29 ± 8.04	9.53 ± 2.94	36.35 ± 2.16
ZINC12007063	/	/	78.48 ± 2.16	63.22 ± 2.83	66.18 ± 2.16	61.18 ± 3.27	17.29 ± 3.56	24.35 ± 0.82	16.21 ± 1.41	63.94 ± 3.56	36.27 ± 2.16	13.02 ± 1.63
Atrazine	/	/	96.54 ± 1.63	92.45 ± 4.32	92.62 ± 4.32	90.23 ± 2.83	/	/	/	/	/	/
Azoxystrobin	/	/	/	/	/	/	56.49 ± 1.41	71.29 ± 1.63	75.19 ± 2.16	100	88.10 ± 1.41	84.34 ± 2.45

Note: Representative fungi used in this study included *B. cinereal*, *C. arachidicola*, *G. zeae*, *P. sasakii*, *S. sclerotiorum*, and *R. cerealis*; “/” means that which is not detected or non-existent.

## Supplementary Materials

The following are available online, Figure S1: The binding mode between the small molecules and transketolase, Figure S2: Molecular structure of compound 4b shown as thermal, Table S1: The Docking affinity of the compounds with transketolase, the other supplementary database: The appearance, yields, melting points and spectral data of the synthesized compounds.
