# Supplementary material for: Structure-Based Discovery and Synthesis of Potential Transketolase Inhibitors
Source: Molecules. 2018 Aug 23;23(9):2116. doi: 10.3390/molecules23092116 (PMC6225308; doi:10.3390/molecules23092116)
Supplement: Supplementary file 1 [file molecules-23-02116-s001.pdf]

molecules

Supplementary Materials

Structure-Based Discovery and Synthesis of Potential Transketolase Inhibitors

Jingqian Huo [1,†], Bin Zhao [2,†], Zhe Zhang [1], Jihong Xing [3], Jinlin Zhang [1,*], Jingao Dong [1,3,*] and Zhijin Fan [2,4,*]

1 College of Plant Protection, Agricultural University of Hebei, Baoding 071001, China; huojingqian@163.com (J.H.); zhang_z2013@163.com (Z.Z.)
2 State Key Laboratory of Elemento-Organic Chemistry, College of Chemistry, Nankai University, Tianjin 300071, P. R. China; bdzhaobin@126.com
3 College of life science, Agricultural University of Hebei, Baoding 071000, China; xingjihong2000@126.com
4 Collaborative Innovation Center of Chemical Science and Engineering (Tianjin), Nankai University, Tianjin 300071, China
* Correspondence: zhangjinlin@hebau.edu.cn (J.Z.); dongjingao@126.com (J.D.); fanzj@nankai.edu.cn (Z.F.)
† These authors contributed equally to this work.

Table of Contents

Table S1. The docking affinity of the compounds with transketolase.
Figure S1. The binding mode between the small molecules and transketolase.
Figure S2. Molecular structure of compound **4b**.
The appearance, yields, melting points and spectral data of the synthesized compounds.

Table S1. The docking affinity of the compounds with transketolase

ZINC Compd.	Affinity (kcal/mol)	ZINC Compd.	Affinity (kcal/mol)	ZINC Compd.	Affinity (kcal/mol)	ZINC Compd.	Affinity (kcal/mol)	ZINC Compd.	Affinity (kcal/mol)
12007063	-9.1	05776152	-8.7	12929396	-8.6	67603588	-8.5	41585313	-8.5
19961402	-9	72020240	-8.7	95353804	-8.6	72472311	-8.5	46127235	-8.5
12126699	-9	16283531	-8.7	95353805	-8.6	72472321	-8.5	49169766	-8.5
23186631	-8.8	08654961	-8.6	95381421	-8.6	72472332	-8.5	05175849	-8.5
12130512	-8.8	58191888	-8.6	12130884	-8.6	21941633	-8.5	19706239	-8.5

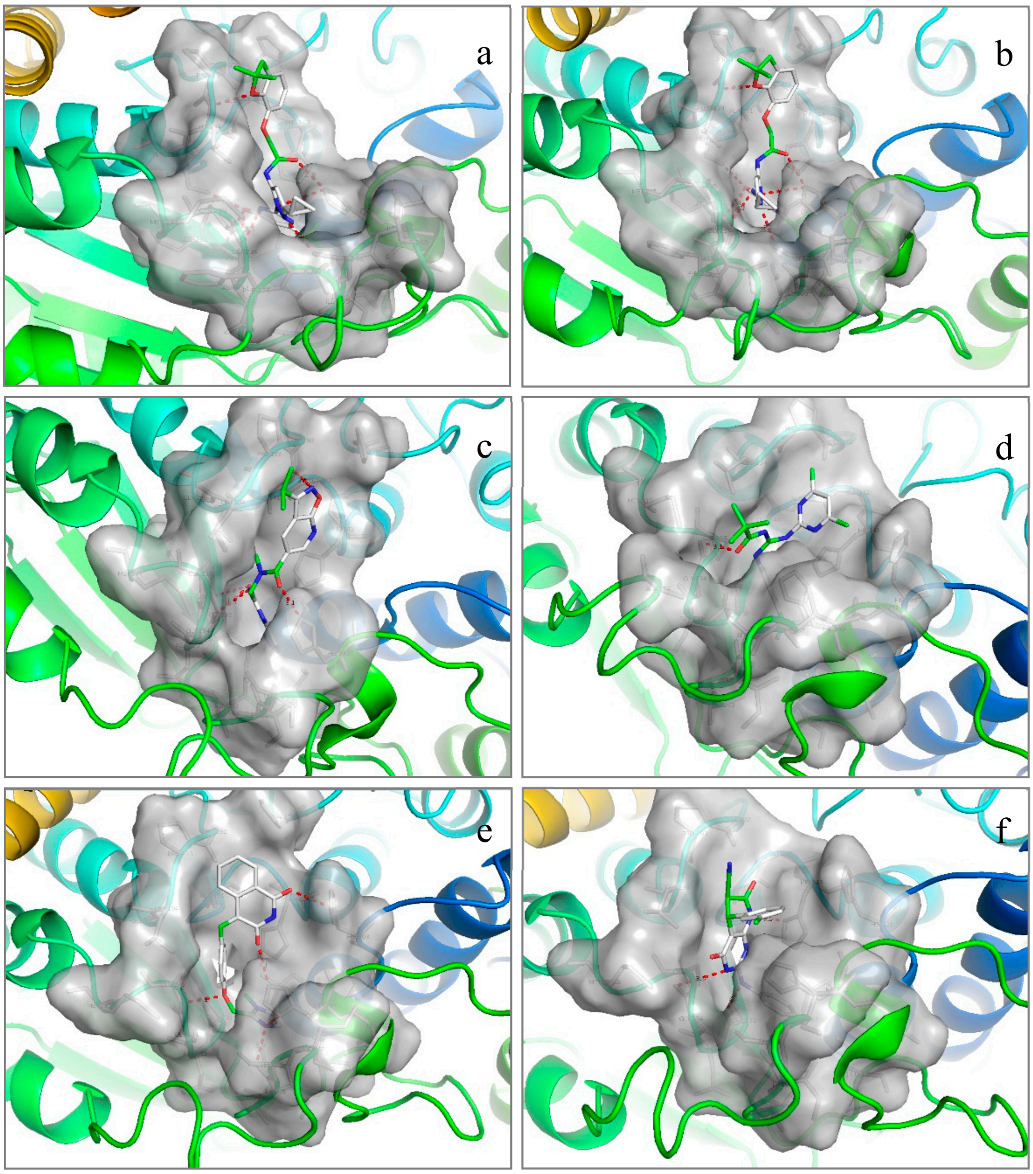

Figure S1. The binding mode between the small molecules and transketolase. (a) ZINC12007063; (b) ZINC12126699; (c) ZINC12929396; (d) ZINC16283531; (e) ZINC58191888; (f) ZINC19961402.

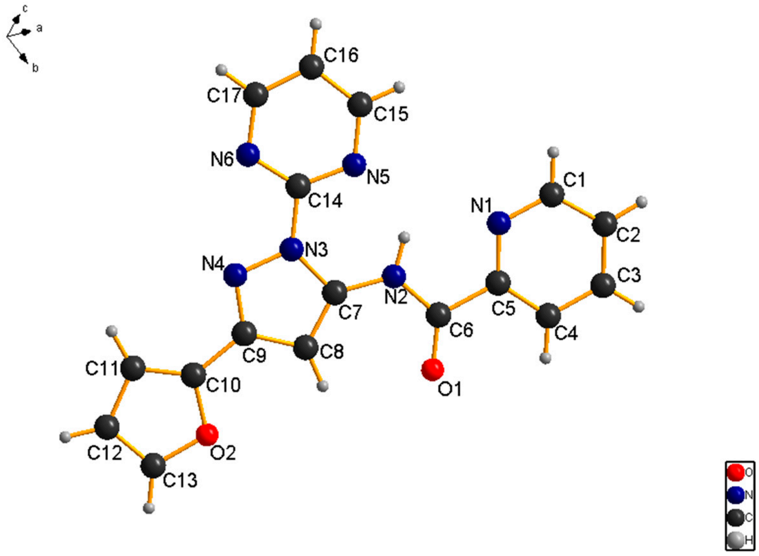

Figure S2. Molecular structure of compound **4b.**

The appearance, yields, melting points and spectral data of the synthesized compounds

2-((2,2-dimethyl-2,3-dihydrobenzofuran-7-yl)oxy)-N-(3-(furan-2-yl)-1-(pyrimidin-2-yl)-1H-pyrazol-5-yl)acetamide (**4a**). Yellow solid, yield 85.53%, M.p.: 200-202 ℃, ^{1}H-NMR (400 MHz, $CDCl_3$) δ=12.83 (s, 1H, NH), 8.83 (d, *J* = 4.8 Hz, 2H, pyrimidine-H), 7.54 (d, *J* = 1.1 Hz, 1H, furan-H), 7.37 (s, 1H, pyrazole-H), 7.19 (t, *J* = 4.8 Hz, 1H, pyrimidine-H), 6.99 (d, *J* = 3.3 Hz, 1H, furan-H), 6.88 (dd, *J* = 7.3, 4.9 Hz, 1H, Ph-H), 6.86 – 6.79 (m, 2H, Ph-H), 6.53 (dd, *J* = 3.3, 1.8 Hz, 1H, furan-H), 4.82 (s, 2H, $-OCH_2-$), 3.09 (s, 2H, CH_2), 1.55 (s, 6H, $2{\times}CH_3$); ^{13}C-NMR (100 MHz, $CDCl_3$): δ = 166.11, 158.62, 157.40, 147.70, 146.65, 142.96, 142.21, 140.24, 129.48, 120.87, 120.58, 119.42, 117.68, 114.00, 111.56, 108.71, 95.84, 88.05, 68.86, 43.17, 28.27; HRMS(ESI)[M+H]$^+$ calcd for $C_{23}H_{21}N_5O_4$: 432.1672, found : 432.1667.

N-(3-(furan-2-yl)-1-(pyrimidin-2-yl)-1H-pyrazol-5-yl)picolinamide (**4b).** Yellow solid, yield 84.96%, M.p.: 210-212 ℃, ^{1}H-NMR (400 MHz, $CDCl_3$) δ=13.68 (s, 1H, NH), 8.93 (d, *J* = 4.8 Hz, 2H, pyrimidine-H), 8.73 (d, *J* = 4.0 Hz, 1H, pyridine-H), 8.33 (d, *J* = 7.8 Hz, 1H, pyridine-H), 7.95 (t, *J* = 7.3 Hz, 1H, pyrimidine-H), 7.55 (s, 2H, pyridine-H), 7.50 (s, 1H, pyrazole-H), 7.28 (d, *J* = 2.5 Hz, 1H, furan-H), 7.00 (d, *J* = 3.3 Hz, 1H, furan-H), 6.53 (dd, *J* = 3.3, 1.7 Hz, 1H, furan-H); ^{13}C-NMR (100 MHz, $CDCl_3$): δ = 161.79, 158.84, 157.77, 149.94, 149.15, 148.02, 146.88, 143.04, 141.03, 137.77, 127.01, 123.10, 118.05, 111.67, 108.72, 95.79; HRMS(ESI)[M+H]$^+$ calcd for $C_{17}H_{12}N_6O_2$: 333.1100, found : 333.1098.

N-(3-(furan-2-yl)-1-(pyrimidin-2-yl)-1H-pyrazol-5-yl)nicotinamide (**4c**). White solid, yield 72.81%, M.p.: 248-250 ℃, ^{1}H-NMR (400 MHz, $CDCl_3$) δ 12.79 (s, 1H, NH), 9.24 (d, *J* = 2.0 Hz, 1H, pyridine-H), 8.89 (d, *J* = 4.9 Hz, 2H, pyrimidine-H), 8.84 (dd, *J* = 4.8, 1.6 Hz, 1H, pyridine-H), 8.40 – 8.36 (m, 1H, pyridine-H), 7.56-7.53 (m, 1H, pyridine-H), 7.52 (d, *J* = 4.9 Hz, 1H, furan-H), 7.44 (s, 1H, pyrazolee-H), 7.30 (t, *J* = 4.9 Hz, 1H, pyrimidine-H), 6.99 (d, *J* = 3.4 Hz, 1H, furan-H), 6.52 (dd, *J* = 3.4, 1.8 Hz, 1H, furan-H); ^{13}C-NMR (100 MHz, $CDCl_3$): δ = 161.35, 158.64, 157.60, 153.02, 147.90, 147.47, 146.81, 143.04, 140.74, 135.79, 129.39, 123.96, 117.96, 111.55, 108.89, 95.95;

HRMS(ESI)[M+H]+ calcd for $C_{17}H_{12}N_6O_2$: 333.1100, found : 333.1099.

N-(3-(furan-2-yl)-1-(pyrimidin-2-yl)-1H-pyrazol-5-yl)isonicotinamide(**4d**). Yellow solid, yield 70.67%, M.p.: 274-276 °C, ^{1}H-NMR (400 MHz, $CDCl_3$) δ=12.80 (s, 1H, NH), 8.90 (d, *J* = 4.8 Hz, 4H, pyrimidine-H and pyridine-H), 7.85 (d, *J* = 4.6 Hz, 2H, pyridine-H), 7.56 (s, 1H, furan-H), 7.46 (s, 1H, pyrazolee-H), 7.32 (t, *J* = 4.7 Hz, 1H, pyrimidine-H), 7.01 (d, *J* = 2.8 Hz, 1H, furan-H), 6.54 (s, 1H, furan-H); ^{13}C-NMR (100 MHz, $CDCl_3$): δ = 160.39, 157.67, 156.56, 150.00, 146.40, 145.84, 142.09, 139.67, 139.54, 119.89, 117.02, 110.56, 107.94, 95.16; HRMS(ESI)[M+H]+ calcd for $C_{17}H_{12}N_6O_2$: 333.1100, found : 333.1101.

N-(3-(furan-2-yl)-1-(pyrimidin-2-yl)-1H-pyrazol-5-yl)-6-(trifluoromethyl)picolinamide(**4e**). White solid, yield 89.93%, M.p.: 315-317 °C, ^{1}H-NMR (400 MHz, $CDCl_3$) δ13.74 (s, 1 H, NH), 9.01 (s, 1 H, pyridine-H), 8.92 (d, *J* =4.8 Hz, 2 H, pyrimidine-H), 8.47 (d, *J* =8.1 Hz, 1 H, pyridine-H), 8.21 (dd, *J* =8.2, 1.7 Hz, 1 H, pyridine-H), 7.55 (d, *J* =1.1 Hz, 1 H, furan-H), 7.48 (s, 1 H, pyrazolee-H), 7.30 (d, *J* =4.8 Hz, 1 H, pyrimidine-H), 7.00 (d, *J* =3.2 Hz, 1 H, furan-H), 6.54 (dd, *J* =3.3, 1.8 Hz, 1 H, furan-H); ^{13}C-NMR (100 MHz, $CDCl_3$): δ = 160.11, 158.65, 157.51, 152.16, 147.64, 146.68, 145.76, 142.94, 140.37, 135.06, 129.45, 129.12, 122.80, 117.98, 111.52, 108.65, 95.97; HRMS(ESI)[M+H]+ calcd for $C_{18}H_{12}ClN_5O_2$: 401.0974, found : 401.0972.

N-(3-(furan-2-yl)-1-(pyrimidin-2-yl)-1H-pyrazol-5-yl)thiophene-2-carboxamide(**4f**). Yellow solid, yield 63.60%, M.p.: 221-223 °C, ^{1}H-NMR (400 MHz, $CDCl_3$) δ 12.56 (s, 1H, NH), 8.88 (d, *J* = 4.8 Hz, 2H, pyrimidine-H), 7.75 (d, *J* = 3.7 Hz, 1H, thiophene-H), 7.62 (d, *J* = 4.9 Hz, 1H, thiophene-H), 7.54 (s, 1H, furan-H), 7.36 (s, 1H, pyrazolee-H), 7.27 (d, *J* = 4.8 Hz, 1H, thiophene-H), 7.21 – 7.17 (m, 1H, pyrimidine-H), 6.98 (d, *J* = 3.3 Hz, 1H, furan-H), 6.52 (s, 1H, furan-H); ^{13}C-NMR (100 MHz, $CDCl_3$): δ = 158.56, 157.65, 147.60, 146.89, 143.01, 140.90, 134.71, 133.65, 131.49, 129.41, 128.12, 117.84, 111.51, 108.85, 95.54; HRMS(ESI)[M+H]+ calcd for $C_{16}H_{11}N_5O_2S$: 338.0712, found : 338.0708.

N-(3-(furan-2-yl)-1-(pyrimidin-2-yl)-1H-pyrazol-5-yl)furan-2-carboxamide (**4g**). Yellow solid, yield 57.52%, M.p.: 150-152 °C, ^{1}H-NMR (400 MHz, $CDCl_3$) δ 12.65 (s, 1H, NH), 8.90 (d, *J* = 4.8 Hz, 2H, pyrimidine-H), 7.63 (s, 1H, furan-H), 7.55 (s, 1H, furan-H), 7.40 (s, 1H, pyrazolee-H), 7.35 (d, *J* = 3.1 Hz, 1H, furan-H), 7.28 (d, *J* = 4.6 Hz, 1H, pyrimidine-H), 7.00 (d, *J* = 3.1 Hz, 1H, furan-H), 6.64 (d, *J* = 1.8 Hz, 1H, furan-H), 6.54 (s, 1H, furan-H); ^{13}C-NMR (100 MHz, $CDCl_3$): δ = 158.57, 157.65, 154.35, 147.65, 147.50, 146.83, 144.85, 142.96, 140.51, 117.82, 116.16, 112.82, 111.49, 108.75, 95.65; HRMS(ESI)[M+H]+ calcd for $C_{16}H_{11}N_5O_3$: 322.0940, found : 322.0940.

N-(3-(furan-2-yl)-1-(pyrimidin-2-yl)-1H-pyrazol-5-yl)benzamide(**4h**). Yellow solid, yield 89.30%, M.p.: 225-227 °C, ^{1}H-NMR (400 MHz, $CDCl_3$) δ 12.64 (s, 1H, NH), 8.90 (d, *J* = 4.8 Hz, 2H, pyrimidine-H), 8.04 (d, *J* = 7.3 Hz, 2H, Ph-H), 7.65 (t, *J* = 7.2 Hz, 1H, Ph-H), 7.59 (d, *J* = 7.6 Hz, 2H, Ph-H), 7.56(s, 1H, furan-H), 7.47 (s, 1H, pyrazolee-H), 7.31 (s, 1H, pyrimidine-H), 7.00 (d, *J* = 3.2 Hz, 1H, furan-H), 6.54 (d, *J* = 1.4 Hz, 1H, furan-H); ^{13}C-NMR (100 MHz, $CDCl_3$): δ = 163.28, 158.52, 157.51, 147.65, 146.69, 142.92, 141.25, 133.42, 132.44, 128.94, 127.22, 117.79, 111.53, 108.74, 95.48; HRMS(ESI)[M+H]+ calcd for $C_{18}H_{13}N_5O_2$: 332.1147, found : 332.1149.

N-(3-(furan-2-yl)-1-(pyrimidin-2-yl)-1H-pyrazol-5-yl)-2-(trifluoromethyl)benzamide(**4i**). White solid,

yield 83.00%, M.p.: 256-258 °C, ^{1}H-NMR (400 MHz, $CDCl_3$) δ 12.14 (s, 1H, NH), 8.74 (d, *J* = 4.9 Hz, 2H, pyrimidine-H), 7.84 (d, *J* = 6.9 Hz, 1H, Ph-H), 7.75 (d, *J* = 7.3 Hz, 1H, Ph-H), 7.73 (s, 1H, Ph-H), 7.71 (d, *J* = 1.8 Hz, 1H, Ph-H), 7.55 (d, *J* = 1.1 Hz, 1H, furan-H), 7.49 (s, 1H, pyrazolee-H), 7.21 (t, *J* = 4.9 Hz, 1H, pyrimidine-H), 7.00 (d, *J* = 3.3 Hz, 1H, furan-H), 6.54 (dd, *J* = 3.4, 1.8 Hz, 1H, furan-H); ^{13}C-NMR (100 MHz, $CDCl_3$): δ = 163.68, 158.51, 157.54, 147.60, 146.73, 143.03, 140.48, 135.19, 132.36, 130.79, 128.74, 126.87, 124.87, 122.15, 117.89, 111.55, 108.87, 96.26; HRMS(ESI)$[M+H]^+$ calcd for $C_{19}H_{12}F_3N_5O_2$: 400.1021, found : 400.1020.

N-(3-(furan-2-yl)-1-(pyrimidin-2-yl)-1H-pyrazol-5-yl)-3-(trifluoromethyl)benzamide (**4j**). White solid, yield 83.00%, M.p.: 186-188 °C, ^{1}H-NMR (400 MHz, $CDCl_3$) δ 12.89 (s, 1H, NH), 8.89 (d, *J* = 4.9 Hz, 2H, pyrimidine-H), 8.28 (d, *J* = 8.6 Hz, 2H, Ph-H), 7.90 (d, *J* = 7.6 Hz, 1H, Ph-H), 7.74 (t, *J* = 7.7 Hz, 1H, Ph-H), 7.56 (d, *J* = 1.1 Hz, 1H, furan-H), 7.45 (s, 1H, pyrazolee-H), 7.33 (t, *J* = 4.9 Hz, 1H, pyrimidine-H), 7.01 (d, *J* = 3.1 Hz, 1H, furan-H), 6.54 (dd, *J* = 3.4, 1.8 Hz, 1H, furan-H); ^{13}C-NMR (100 MHz, $CDCl_3$): δ = 161.58, 158.53, 157.49, 147.47, 146.76, 143.02, 140.87, 134.23, 130.92, 129.79, 128.94, 125.01, 123.87, 122.30, 117.97, 111.55, 108.89, 95.66; HRMS(ESI)$[M+H]^+$ calcd for $C_{19}H_{12}F_3N_5O_2$: 400.1021, found : 400.1023.

N-(3-(furan-2-yl)-1-(pyrimidin-2-yl)-1H-pyrazol-5-yl)-4-(trifluoromethyl)benzamide(**4k**). Yellow solid, yield 80.80%, M.p.: 244-246 °C, ^{1}H-NMR (400 MHz, $CDCl_3$) δ 12.75 (s, 1H, NH), 8.90 (d, *J* = 4.8 Hz, 2H, pyrimidine-H), 8.15 (d, *J* = 8.1 Hz, 2H, Ph-H), 7.86 (d, *J* = 8.1 Hz, 2H, Ph-H), 7.57 (s, 1H, furan-H), 7.47 (s, 1H, pyrazolee-H), 7.32 (t, *J* = 4.9 Hz, 1H, pyrimidine-H), 7.02 (d, *J* = 3.0 Hz, 1H, furan-H), 6.55 (d, *J* = 1.6 Hz, 1H, furan-H); ^{13}C-NMR (100 MHz, $CDCl_3$): δ = 161.90, 158.54, 157.52, 147.45, 146.74, 143.01, 140.82, 136.81, 127.74, 126.01, 124.91, 122,20, 117.88, 111.55, 108.87, 95.82; HRMS(ESI)$[M+H]^+$ calcd for $C_{19}H_{12}F_3N_5O_2$: 400.1021, found : 400.1018.

3-fluoro-N-(3-(furan-2-yl)-1-(pyrimidin-2-yl)-1H-pyrazol-5-yl)benzamide(**4l**). Yellow solid, yield 90.35%, M.p.: 196-198 °C, ^{1}H-NMR (400 MHz, $CDCl_3$) δ 12.68 (s, 1H, NH), 8.90 (d, *J* = 4.8 Hz, 2H, pyrimidine-H), 7.81 (d, *J* = 7.7 Hz, 1H, Ph-H), 7.73 (d, *J* = 9.2 Hz, 1H, Ph-H), 7.58 (d, *J* = 8.1 Hz, 1H, furan-H), 7.55 (s, 1H, Ph-H), 7.44 (s, 1H, pyrazolee-H), 7.35 (d, *J* = 6.0 Hz, 1H, Ph-H), 7.31 (d, *J* = 4.8 Hz, 1H, pyrimidine-H), 7.00 (d, *J* = 3.3 Hz, 1H, furan-H), 6.55 – 6.52 (m, 1H, furan-H); ^{13}C-NMR (100 MHz, $CDCl_3$): δ = 164.30, 162.10, 161.82, 158.73, 157.72, 147.69, 146.96, 143.15, 141.11, 130.86, 130.79, 119.63, 118.00, 114.79, 111.67, 108.99, 95.88; HRMS(ESI)$[M+H]^+$ calcd for $C_{18}H_{12}FN_5O_2$: 350.1053, found : 350.1055.

4-fluoro-N-(3-(furan-2-yl)-1-(pyrimidin-2-yl)-1H-pyrazol-5-yl)benzamide (**4m**). Yellow solid, yield 82.00%, M.p.: 250-252 °C, ^{1}H-NMR (400 MHz, $CDCl_3$) δ 12.59 (s, 1H, NH), 8.88 (d, *J* = 4.8 Hz, 2H, pyrimidine-H), 8.04 (dd, *J* = 8.7, 5.2 Hz, 2H, Ph-H), 7.55 (d, *J* = 1.0 Hz, 1H, furan-H), 7.43 (s, 1H, pyrazolee-H), 7.28 (dd, *J* = 5.2, 2.7 Hz, 2H, Ph-H), 7.23 (s, 1H, pyrimidine-H), 6.99 (d, *J* = 3.3 Hz, 1H, furan-H), 6.53 (dd, *J* = 3.3, 1.7 Hz, 1H, furan-H); ^{13}C-NMR (100 MHz, $CDCl_3$): δ = 166.56, 164.03, 162.29, 158.60, 157.68, 147.59, 146.90, 143.02, 141.18, 129.76, 129.67, 117.81, 116.25, 116.03, 111.52, 108.86, 95.63; HRMS(ESI)$[M+H]^+$ calcd for $C_{18}H_{12}FN_5O_2$: 350.1053, found : 350.1054.

2-fluoro-N-(3-(furan-2-yl)-1-(pyrimidin-2-yl)-1H-pyrazol-5-yl)benzamide (**4n**). White solid, yield 87.42%, M.p.: 214-216 °C, ^{1}H-NMR (400 MHz, $CDCl_3$) δ 12.97 (d, *J* = 10.7 Hz, 1H, NH), 8.86 (d, *J*

= 4.8 Hz, 2H, pyrimidine-H), 8.28 (t, *J* = 7.9 Hz, 1H, Ph-H), 7.60 (d, *J* = 6.6 Hz, 1H, Ph-H), 7.55 (s, 1H, furan-H), 7.52 (s, 1H, Ph-H), 7.37 (t, *J* = 7.6 Hz, 1H, pyrimidine-H), 7.27 (s, 1H, pyrazole-H), 7.24 (dd, *J* = 9.6, 5.6 Hz, 1H, Ph-H), 7.00 (d, *J* = 3.0 Hz, 1H, furan-H), 6.55 – 6.52 (m, 1H, furan-H); ^{13}C-NMR (100 MHz, $CDCl_3$): δ = 162.06, 158.57, 157.53, 147.96, 146.78, 143.04, 141.04, 134.48, 132.70, 125.22, 118.07, 116.45, 111.68, 108.76, 96.79; HRMS(ESI)[M+H]$^+$ calcd for $C_{18}H_{12}FN_5O_2$: 350.1053, found : 350.1054.

2-chloro-N-(3-(furan-2-yl)-1-(pyrimidin-2-yl)-1H-pyrazol-5-yl)benzamide (**4o**). White solid, yield 87.92%, M.p.: 252-254 °C, ^{1}H-NMR (400 MHz, $CDCl_3$) δ12.46 (s, 1 H, NH), 8.78 (d, *J* =4.9 Hz, 2 H, pyrimidine-H), 7.85 (dd, *J* =7.5, 1.7 Hz, 1 H, Ph-H), 7.55 – 7.51 (m, 1 H, furan-H), 7.49 (ddd, *J* =7.0, 3.6, 1.1 Hz, 2 H, Ph-H), 7.45 (dd, *J* =4.3, 1.9 Hz,1 H, Ph-H), 7.42 (s, 1 H, pyrazole-H), 7.22 (t, *J* =4.9 Hz, 1 H, pyrimidine-H), 6.98 (dd, *J* =3.3, 0.5 Hz, 1 H, furan-H), 6.52 (dd, *J* =3.4, 1.8 Hz, 1 H, furan-H); ^{13}C-NMR (100 MHz, $CDCl_3$): δ = 162.55, 158.52, 157.50, 147.62, 146.76, 142.99, 140.58, 134.40, 132.22, 131.00, 130.95, 130.73, 127.40, 117.89, 111.50, 108.78, 96.31; HRMS(ESI)[M+H]$^+$ calcd for $C_{18}H_{12}ClN_5O_2$: 366.0758, found :366.0759.

3-chloro-N-(3-(furan-2-yl)-1-(pyrimidin-2-yl)-1H-pyrazol-5-yl)benzamide (**4p**). White solid, yield 89.23%, M.p.: 248-250 °C, ^{1}H-NMR (400 MHz, $CDCl_3$) δ12.70 (s, 1 H, NH), 8.88 (d, *J* =4.9 Hz, 2 H, pyrimidine-H), 8.00 (t, *J* =1.8 Hz, 1 H, Ph-H), 7.93 – 7.88 (m, 1 H, Ph-H), 7.60 (ddd, *J* = 8.0, 1.9, 1.0 Hz, 1 H, Ph-H), 7.55 (d, *J* =1.1 Hz, 1 H, furan-H), 7.51 (t, *J* =7.9 Hz, 1 H, Ph-H), 7.42 (s, 1 H, pyrazole-H), 7.30 (d, *J* =4.9Hz, 1 H, pyrimidine-H), 7.01 – 6.98 (m, 1 H, furan-H), 6.53 (dd, *J* =3.4, 1.8 Hz, 1 H, furan-H); ^{13}C-NMR (100 MHz, $CDCl_3$): δ = 161.86, 158.62, 157.63, 147.54, 146.87, 143.02, 140.97, 135.35, 135.15, 132.42, 130.32, 127.66, 125.40, 117.88, 111.53, 108.88, 95.74; HRMS(ESI)[M+H]$^+$ calcd for $C_{18}H_{12}ClN_5O_2$: 366.0758, found : 366.0757.

4-chloro-N-(3-(furan-2-yl)-1-(pyrimidin-2-yl)-1H-pyrazol-5-yl)benzamide (**4q**). White solid, yield 89.28%, M.p.: 232-234 °C, ^{1}H-NMR (400 MHz, $CDCl_3$) δ12.61 (s, 1 H, NH), 8.87 (d, *J* =4.9 Hz, 2 H, pyrimidine-H), 7.98 – 7.93 (m, 2 H, Ph-H), 7.55 (d, *J* =1.9 Hz, 1 H, furan-H), 7.54 – 7.52 (m, 2 H, Ph-H), 7.42 (s, 1 H, pyrazole-H), 7.28 (t, *J* =4.9 Hz,1 H, pyrimidine-H), 6.98 (d, *J* =3.4 Hz, 1 H, furan-H), 6.52 (dd, *J* =3.4, 1.8 Hz, 1 H, furan-H); ^{13}C-NMR (100 MHz, $CDCl_3$): δ = 162.18, 158.52, 157.54, 147.54, 146.74, 142.97, 141.04, 138.76, 131.94, 129.23, 128.65, 117.81, 111.53, 108.81, 95.62; HRMS(ESI)[M+H]$^+$ calcd for $C_{18}H_{12}ClN_5O_2$: 366.0758, found :366.0759.

2,6-difluoro-N-(3-(furan-2-yl)-1-(pyrimidin-2-yl)-1H-pyrazol-5-yl)benzamide (**4r**). Yellow solid, yield 79.41%, M.p.: 206-208 °C, ^{1}H-NMR (400 MHz, $CDCl_3$) δ12.45 (s, 1 H, NH), 8.79 (d, *J* =4.8 Hz, 2 H, pyrimidine-H), 7.52 (d, *J* =5.6Hz, 1 H, Ph-H), 7.49 (d, *J* =4.9Hz, 1 H, furan-H), 7.27 (s, 1 H, pyrazole-H), 7.23 (t, *J* =4.4Hz, 1 H, pyrimidine-H), 7.07 (t, *J* =8.9Hz, 2 H, Ph-H), 6.97 (d, *J* =2.7Hz, 1 H, furan-H), 6.54 – 6.50 (m, 1 H, furan-H); ^{13}C-NMR (100 MHz, $CDCl_3$): δ = 161.90, 159.37, 158.46, 157.38, 156.25, 147.55, 146.60, 142.96, 140.24, 132.99, 117.94, 112.51, 111.51, 108.76, 96.46; HRMS(ESI)[M+H]$^+$ calcd for $C_{18}H_{11}F_2N_5O_2$: 368.0959, found : 368.0962.

2,4-dichloro-N-(3-(furan-2-yl)-1-(pyrimidin-2-yl)-1H-pyrazol-5-yl)benzamide (**4s**). White solid, yield 88.92%, M.p.: 283-285 °C, ^{1}H-NMR (400 MHz, $CDCl_3$) δ12.52 (s, 1 H, NH), 8.80 (d, *J* =4.9 Hz, 2 H,

pyrimidine-H), 7.84 (d, *J* =8.4 Hz, 1 H, furan-H), 7.56 (d, *J* =1.8 Hz, 2 H, Ph-H), 7.49 (s, 1 H, pyrazole-H), 7.44 (dd, *J* =8.4, 2.0 Hz, 1 H, Ph-H), 7.26 (t, *J* =4.9 Hz, 1 H, pyrimidine-H), 7.00 (d, *J* =3.4 Hz,1 H, furan-H), 6.54 (dd, *J* =3.4, 1.8 Hz, 1 H, furan-H); ^{13}C-NMR (100 MHz, $CDCl_3$): δ = 161.53, 158.53, 157.49, 147.55, 146.81, 143.04, 140.39, 137.85, 132.81, 132.09, 131.83, 130.54, 127.87, 117.94, 111.52, 108.84, 96.46; HRMS(ESI)[M+H]$^+$ calcd for $C_{18}H_{11}Cl_2N_5O_2$: 400.0368, found :400.0370.

N-(3-(furan-2-yl)-1-(pyrimidin-2-yl)-1H-pyrazol-5-yl)-2-phenoxyacetamide (**4t**). Yellow solid, yield 83.15%, M.p.: 158-160 °C, ^{1}H-NMR (400 MHz, $CDCl_3$) δ12.63 (s, 1 H, NH), 8.73 (d, *J* =4.9 Hz, 2 H, pyrimidine-H), 7.56 – 7.52 (m, 1 H, furan-H), 7.40 (d, *J* =7.6 Hz, 2 H, Ph-H), 7.37(s, 1 H, pyrazole-H), 7.22 (t, *J* =4.9 Hz, 1 H, pyrimidine-H), 7.08 (t, *J* =7.4 Hz, 1 H, Ph-H), 7.04 (d, *J* =8.0 Hz, 2 H, Ph-H), 6.98 (d, *J* =3.4 Hz, 1 H, furan-H), 6.52 (dd, *J* =3.3, 1.8 Hz, 1 H, furan-H), 4.76 (s, 2 H, CH_2); ^{13}C-NMR (100 MHz, $CDCl_3$): δ = 165.53, 158.39, 157.38, 157.25, 147.65, 146.58, 142.94, 140.03, 130.00, 122.40, 117.94, 114.55, 111.55, 108.69, 95.84, 67.48; HRMS(ESI)[M+H]$^+$ calcd for $C_{19}H_{15}N_5O_3$: 362.1253, found : 362.1253.

2-(2,4-dichlorophenoxy)-N-(3-(furan-2-yl)-1-(pyrimidin-2-yl)-1H-pyrazol-5-yl)acetamide (**4u**). White solid, yield 88.96%, M.p.: 255-257 °C, ^{1}H-NMR (400 MHz, $CDCl_3$) δ12.41 (s, 1 H, NH), 8.71 (d, *J* =4.7 Hz, 2 H, pyrimidine-H), 7.44 (s, 1 H, Ph-H), 7.39 (s, 1 H, pyrazole-H), 7.19 (d, *J* =8.2 Hz, 1 H, furan-H), 7.15 (d, *J* =4.1 Hz, 1 H, Ph-H), 7.13 (d, *J* =4.7 Hz, 1 H, pyrimidine-H), 6.89 (d, *J* =2.7 Hz, 1 H, Ph-H), 6.85 (d, *J* =8.8 Hz, 1 H, furan-H), 6.43 (s, 1 H, furan-H), 4.69 (d, *J* =13.7 Hz, 2 H, CH_2); ^{13}C-NMR (100 MHz, $CDCl_3$): δ = 163.40, 157.56, 156.41, 150.66, 146.53, 145.57, 141.93, 138.67, 129.48, 127.23, 126.80, 122.55, 116.95, 113.65, 110.48, 107.66, 95.28, 67.67; HRMS(ESI)[M+H]$^+$ calcd for $C_{19}H_{13}Cl_2N_5O_3$: 430.0474, found : 430.0472.

N-(3-(furan-2-yl)-1-(pyrimidin-2-yl)-1H-pyrazol-5-yl)-4-nitrobenzamide(**4v**). Yellow solid, yield 76.15%, M.p.: 182-184 °C, ^{1}H-NMR (400 MHz, DMSO) δ12.70 (s, 1 H, NH), 9.06 (d, *J* =4.9 Hz, 2 H, pyrimidine-H), 8.46 (d, *J* =8.8 Hz, 2 H, Ph-H), 8.26 (d, *J* =8.9 Hz, 2 H, Ph-H), 7.85 (d, *J* =1.1 Hz, 1 H, furan-H), 7.60 (t, *J* =4.9 Hz, 1 H, pyrimidine-H), 7.27 (s, 1 H, pyrazole-H), 7.06 (d, *J* =2.8 Hz, 1 H, furan-H), 6.67 (dd, *J* =3.4, 1.8 Hz, 1 H, furan-H); ^{13}C-NMR (100 MHz, DMSO): δ = 161.69, 159.70, 148.50, 147.73, 145.22, 144.19, 140.62, 138.86, 129.44, 124.81, 119.61, 112.42, 109.01, 99.99, 95.61; HRMS(ESI)[M+H]$^+$ calcd for $C_{18}H_{12}ClN_5O_2$: 377.0998, found :377.0999.

N-(3-(furan-2-yl)-1-(pyrimidin-2-yl)-1H-pyrazol-5-yl)-4-methoxybenzamide (**4w**). Yellow solid, yield 71.03%, M.p.: 139-141 °C, ^{1}H-NMR (400 MHz, $CDCl_3$) δ12.50 (s, 1 H, NH), 8.87 (d, *J* =4.8 Hz, 2 H, pyrimidine-H), 7.98 (d, *J* =8.7 Hz, 2 H, Ph-H), 7.53 (s, 1 H, furan-H), 7.42 (s, 1 H, pyrazole-H), 7.26 (d, *J* =4.7 Hz, 1 H, pyrimidine-H), 7.04 (d, *J* =8.7 Hz, 2 H, Ph-H), 6.97 (d, *J* =3.1 Hz, 1 H, furan-H), 6.51 (d, *J* =1.5 Hz, 1 H, furan-H), 3.91 (s, 3 H, OCH_3); ^{13}C-NMR (100 MHz, $CDCl_3$): δ = 162.95, 162.84, 158.52, 157.60, 147.78, 146.73, 142.90, 141.55, 129.16, 125.68, 117.74, 114.16, 111.53, 108.70, 95.23, 55.50; HRMS(ESI)[M+H]$^+$ calcd for $C_{19}H_{15}N_5O_3$: 362.1253, found :362.1247.

N-(3-(furan-2-yl)-1-(pyrimidin-2-yl)-1H-pyrazol-5-yl)cyclopropanecarboxamide (**4x**). Yellow solid, yield 89.05%, M.p.: 194-196 °C, ^{1}H-NMR (400 MHz, $CDCl_3$) δ11.84 (s, 1 H, NH), 8.83 (d, *J* =4.9

Hz, 2 H, pyrimidine-H), 7.52 – 7.48 (m, 1 H, furan-H), 7.24 (t, *J* =4.9 Hz, 2 H, pyrimidine-H, pyrazole-H), 6.91 (d, *J* =3.4 Hz, 1 H, furan-H), 6.49 (dd, *J* =3.4, 1.8 Hz, 1 H, furan-H), 1.67 (dd, *J* =8.3, 3.9 Hz, 1 H, CH), 1.21 – 1.14 (m, 2 H, CH_2), 0.99 – 0.92 (m, 2 H, CH_2); ^{13}C-NMR (100 MHz, $CDCl_3$): δ = 170.42, 158.48, 157.60, 147.74, 146.59, 142.87, 141.08, 117.78, 111.47, 108.61, 95.34, 16.24, 8.74; HRMS(ESI)[M+H]$^+$ calcd for $C_{15}H_{13}N_5O_2$: 296.1147, found :296.1147.

N-(1-(3-chloropyridin-2-yl)-3-(furan-2-yl)-1H-pyrazol-5-yl)-3-(trifluoromethyl)benzamide(**8a**). White solid, yield 79.86%, M.p.: 129-131 °C, ^{1}H-NMR (400 MHz, $CDCl_3$) δ 11.45 (s, 1 H, NH), 8.46 (dd, *J* =4.7, 1.5 Hz, 1 H, pyridine-H), 8.15 (d, *J* =7.9 Hz, 2 H, Ph-H), 8.06 (dd, *J* =8.0, 1.5 Hz, 1 H, pyridine-H), 7.87 (d, *J* =7.8 Hz, 1 H, furan-H), 7.70 (t, *J* =7.7 Hz, 1 H, Ph-H), 7.55 (dd, *J* =1.7, 0.6 Hz, 1 H, Ph-H), 7.35 (dd, *J* =8.0, 4.7 Hz, 1 H, pyridine-H), 7.30 (s, 1 H, pyrazole-H), 6.88 – 6.84 (m, 1 H, furan-H), 6.54 (dd, *J* =3.4, 1.8 Hz, 1 H, furan-H). ^{13}C-NMR (100 MHz, $CDCl_3$): δ = 161.73, 148.76, 147.91, 144.76, 144.57, 142.73, 139.23, 134.21, 131.56, 131.23, 130.68, 129.74, 128.95, 124.88, 123.96, 122.96, 122.25, 111.46, 107.91, 95.22; HRMS(ESI)[M+H]$^+$ calcd for $C_{20}H_{12}ClF_3N_4O_2$: 433.0679, found : 433.0680.

3-chloro-N-(1-(3-chloropyridin-2-yl)-3-(furan-2-yl)-1H-pyrazol-5-yl)benzamide(**8b**). White solid, yield 81.20%, M.p.: 118-120 °C, ^{1}H-NMR (400 MHz, $CDCl_3$) δ 11.10 (s, 1 H, NH), 8.38 (dd, *J* =4.7, 1.5 Hz, 1 H, pyridine-H), 7.94 (dd, *J* =8.0, 1.5 Hz, 1 H, pyridine-H), 7.80 (t, *J* =1.8 Hz, 1 H, Ph-H), 7.67 (d, *J* =7.7 Hz, 1 H, furan-H), 7.49 – 7.45 (m, 1 H, Ph-H), 7.45 (d, *J* =1.1 Hz, 1 H, Ph-H), 7.37 (t, *J* =7.9 Hz, 1 H, Ph-H), 7.24 (dd, *J* = 8.0, 4.7 Hz, 1H, pyridine-H), 7.18 (s, 1 H, pyrazole-H), 6.75 (d, *J* =3.3 Hz, 1 H, furan-H), 6.43 (dd, *J* =3.3, 1.8 Hz, 1 H, furan-H). ^{13}C-NMR (100 MHz, $CDCl_3$): δ = 160.84, 147.64, 146.91, 143.86, 143.49, 141.63, 141.54, 138.17, 134.10, 134.07, 131.33, 129.19, 126.58, 124.08, 123.88, 121.90, 110.39, 106.79, 94.17; HRMS(ESI)[M+H]$^+$ calcd for $C_{19}H_{12}Cl_2N_4O_2$: 399.0416, found : 399.0411.

N-(1-(3-chloropyridin-2-yl)-3-(furan-2-yl)-1H-pyrazol-5-yl)-2-phenoxyacetamide(**8c**). Yellow solid, yield 71.09%, M.p.: 128-130 °C, ^{1}H-NMR (400 MHz, $CDCl_3$) δ 10.95 (s, 1 H, NH), 8.32 (dd, *J* =4.7, 1.6 Hz, 1 H, pyridine-H), 7.96 (dd, *J* =8.0, 1.4 Hz, 1 H, pyridine-H), 7.52 (dd, *J* =1.8, 0.7 Hz, 1 H, furan-H), 7.37 – 7.31 (m, 2 H, Ph-H), 7.27 – 7.24 (m, 1 H, pyridine-H), 7.20 (s, 1 H, pyrazole-H), 7.05 (t, *J* =7.4 Hz, 1 H, Ph-H), 6.92 (dd, *J* =8.7, 0.9 Hz, 2 H, Ph-H), 6.82 (dd, *J* =3.4, 0.6 Hz, 1 H, furan-H), 6.51 (dd, *J* =3.4, 1.8 Hz, 1 H, furan-H), 4.66 (s, 2 H, CH_2). ^{13}C-NMR (100 MHz, $CDCl_3$): δ = 165.19, 156.90, 148.17, 148.03, 145.14, 144.50, 142.61, 142.04, 138.06, 129.91, 125.23, 123.20, 122.40, 114.49, 111.44, 107.67, 95.22, 67.19; HRMS(ESI)[M+H]$^+$ calcd for $C_{20}H_{15}ClN_4O_3$: 395.0911, found : 395.0908.

N-(1-(3-chloropyridin-2-yl)-3-(furan-2-yl)-1H-pyrazol-5-yl)-2-(2,4-dichlorophenoxy)acetamide(**8d**). Yellow solid, yield 81.06%, M.p.: 181-183 °C, ^{1}H-NMR (400 MHz, $CDCl_3$) δ 10.48 (s, 1 H, NH), 8.40 (d, *J* =4.6 Hz, 1 H, pyridine-H), 7.97 (d, *J* =8.0 Hz, 1 H, pyridine-H), 7.50 (s, 1 H, Ph-H), 7.40 (d, *J* =1.8 Hz, 1 H, furan-H), 7.32 – 7.28 (m, 1 H, Ph-H), 7.22 (dd, *J* =8.8, 1.8 Hz, 1 H, pyridine-H), 7.18 (s, 1 H, pyrazole-H), 6.85 (d, *J* =8.8 Hz, 1 H, Ph-H), 6.80 (d, *J* =3.3 Hz, 1 H, furan-H), 6.50 (d, *J* =1.4 Hz, 1 H, furan-H), 4.68 (s, 2 H, CH_2). ^{13}C-NMR (100 MHz, $CDCl_3$): δ = 164.12, 151.36, 148.12, 148.04, 146.04, 144.76, 142.69, 141.83, 137.58, 130.45, 128.21, 127.90, 126.03, 123.63, 123.55, 114.59, 111.47, 107.70, 95.75, 68.29; HRMS(ESI)[M+H]$^+$ calcd for $C_{20}H_{13}Cl_3N_4O_3$: 463.0131, found :

463.0129.

N-(1-(3-chloropyridin-2-yl)-3-(furan-2-yl)-1H-pyrazol-5-yl)-4-methoxybenzamide(**8e**). White solid, yield 53.23%, M.p.: 110-112 ℃, ^{1}H-NMR (400 MHz, $CDCl_3$) δ10.92 (s, 1 H, NH), 8.49 (dd, *J* =4.7, 0.8 Hz, 1 H, pyridine-H), 8.03 (dd, *J* =8.0, 0.8 Hz, 1 H, pyridine-H), 7.86 (d, *J* =8.5 Hz, 2 H, Ph-H), 7.56 – 7.52 (m, 1 H, furan-H), 7.35 – 7.30 (m, 1 H, pyridine-H), 7.27(s, 1 H, pyrazole-H), 7.01 (d, *J* =8.6 Hz, 2 H, Ph-H), 6.83 (d, *J* =3.3 Hz, 1 H, furan-H), 6.55 – 6.50 (m, 1 H, furan-H), 3.90 (s, 3 H, OCH_3). ^{13}C-NMR (100 MHz, $CDCl_3$): δ = 162.95, 162.92, 148.85, 148.13, 144.98, 144.63, 142.63, 142.44, 139.67, 129.11, 125.70, 125.26, 122.88, 114.16, 111.38, 107.76, 94.93, 55.52; HRMS(ESI)$[M+H]^+$ calcd for $C_{20}H_{15}ClN_4O_3$: 395.0911, found : 395.0909.

N-(1-(3-chloropyridin-2-yl)-3-(furan-2-yl)-1H-pyrazol-5-yl)-6-(trifluoromethyl)picolinamide(**8f**). Yellow solid, yield 81.20%, M.p.: 208-210 ℃, ^{1}H-NMR (400 MHz, $CDCl_3$) δ12.05 (s, 1 H, NH), 8.91 – 8.88 (m, 1 H, pyridine-H), 8.56 (dd, *J* =4.7, 1.6 Hz, 1 H, pyridine-H), 8.42 (d, *J* =8.2 Hz, 1 H, pyridine-H), 8.17 (dd, *J* =8.2, 1.8 Hz, 1 H, pyridine-H), 8.01 (dd, *J* =8.0, 1.6 Hz, 1 H, pyridine-H), 7.54 – 7.51 (m, 1 H, furan-H), 7.35 (dd, *J* =8.0, 4.7 Hz, 1 H, pyridine-H), 7.30 (s, 1 H, pyrazole-H), 6.83 (dd, *J* =3.3, 0.5 Hz, 1 H, furan-H), 6.51 (dd, *J* =3.3, 1.8 Hz, 1 H, furan-H). ^{13}C-NMR (100 MHz, $CDCl_3$): δ = 158.66, 150.77, 147.16, 147.01, 144.64, 144.57, 143.65, 141.59, 140.89, 137.38, 134.08, 128.45 128.12, 124.60, 122.39, 121.61, 110.37, 106.62, 94.05; HRMS(ESI)$[M+H]^+$ calcd for $C_{19}H_{11}ClF_3N_5O_2$: 434.0632, found : 434.0626.

N-(1-(3-chloro-5-(trifluoromethyl)pyridin-2-yl)-3-(furan-2-yl)-1H-pyrazol-5-yl)-3-(trifluoromethyl)benzamide (**8g**). White solid, yield 82.39%, M.p.: 96-98 ℃, ^{1}H-NMR (400 MHz, $CDCl_3$) δ 11.38 (s, 1 H, NH), 8.67 (s, 1 H, pyridine-H), 8.27 (d, *J* =1.5 Hz, 1 H, Ph-H), 8.17 (s, 1 H, pyridine-H), 8.11 (d, *J* =7.8 Hz, 1 H, Ph-H), 7.89 (d, *J* =7.7 Hz, 1 H, furan-H), 7.72 (t, *J* =7.8 Hz, 1 H, Ph-H), 7.56 (d, *J* =1.0 Hz, 1 H, Ph-H), 7.33 (s, 1 H, pyrazole-H), 6.88 (d, *J* =3.2 Hz, 1 H, furan-H), 6.54 (dd, *J* =3.2, 1.7 Hz, 1 H, furan-H). ^{13}C-NMR (100 MHz, $CDCl_3$): δ = 160.73, 150.11, 146.42, 144.38, 142.07, 140.55, 139.00, 138.82, 133.07, 130.41, 129.36, 128.78, 128.07, 124.21, 123.55, 123.08, 121.16, 119.77, 110.52, 107.54, 94.78; HRMS(ESI)$[M+H]^+$ calcd for $C_{21}H_{11}ClF_6N_4O_2$: 501.0553, found : 501.0548.

N-(1-(3-chloro-5-(trifluoromethyl)pyridin-2-yl)-3-(furan-2-yl)-1H-pyrazol-5-yl)-2-phenoxyacetamide (**8h**). Yellow solid, yield 68.92%, M.p.: 109-111 ℃, ^{1}H-NMR (400 MHz, $CDCl_3$) δ 11.01 (s, 1 H, NH), 8.40 (d, *J* =1.1 Hz, 1 H, pyridine-H), 8.18 (d, *J* =1.8 Hz, 1 H, pyridine-H), 7.55 (d, *J* =1.1 Hz, 1 H, furan-H), 7.39 – 7.33 (m, 2 H, Ph-H), 7.23 (s, 1 H, pyrazole-H), 7.08 (t, *J* =7.4 Hz, 1 H, Ph-H), 6.97 – 6.92 (m, 2 H, Ph-H), 6.86 (d, *J* =3.3 Hz, 1 H, furan-H), 6.53 (dd, *J* =3.4, 1.8 Hz, 1 H, furan-H), 4.72 (s, 2 H, CH_2). ^{13}C-NMR (100 MHz, $CDCl_3$): δ = 165.45, 156.97, 150.61, 147.60, 145.34, 143.03, 141.88, 139.43, 138.66, 130.05, 125.67, 124.84, 123.60, 122.63, 114.45, 111.54, 108.38, 96.05, 67.34; HRMS(ESI)$[M+H]^+$ calcd for $C_{21}H_{14}ClF_3N_4O_3$: 463.0785, found : 463.0784.

3-chloro-N-(1-(3-chloro-5-(trifluoromethyl)pyridin-2-yl)-3-(furan-2-yl)-1H-pyrazol-5-yl)benzamide (**8i**). White solid, yield 61.03%, M.p.: 195-197 ℃, ^{1}H-NMR (400 MHz, $CDCl_3$) δ11.17 (s, 1 H, NH), 8.71 – 8.67 (m, 1 H, pyridine-H), 8.26 (d, *J* =1.7 Hz, 1 H, pyridine-H), 7.91 (t, *J* =1.8 Hz, 1 H, Ph-H), 7.77 – 7.72 (m, 1 H, furan-H), 7.59 (ddd, *J* =8.0, 2.0, 1.0 Hz, 1 H, Ph-H), 7.55 (dd, *J* =1.7, 0.7

Hz, 1 H, Ph-H), 7.50 (t, *J* =7.9 Hz, 1 H, Ph-H), 7.31 (s, 1 H, pyrazole-H), 6.87 (dd, *J* =3.4, 0.6 Hz, 1 H, furan-H), 6.54 (dd, *J* =3.4, 1.8 Hz, 1 H, furan-H). ^{13}C-NMR (100 MHz, $CDCl_3$): δ = 161.97, 151.10, 147.47, 145.37, 143.07, 141.72, 139.94, 139.85, 135.31, 135.01, 132.58, 130.32, 127.75, 125.50, 124.93, 123.56, 120.85, 111.55, 108.55, 95.82; HRMS(ESI)[M+H]$^+$ calcd for $C_{20}H_{11}Cl_2F_3N_4O_2$: 467.0289, found : 467.0289.

2-chloro-N-(1-(3-chloro-5-(trifluoromethyl)pyridin-2-yl)-3-(furan-2-yl)-1H-pyrazol-5-yl)benzamide (**8j**). White solid, yield 59.06%, M.p.: 142-144 °C, ^{1}H-NMR (400 MHz, $CDCl_3$) δ10.94 (s, 1 H, NH), 8.59 (d, *J* =1.0 Hz, 1 H, pyridine-H), 8.20 (d, *J* =1.7 Hz, 1 H, pyridine-H), 7.90 – 7.85 (m, 1 H, Ph-H), 7.53 (d, *J* =1.1 Hz, 1 H, furan-H), 7.49 – 7.45 (m, 2 H, Ph-H), 7.45 – 7.39 (m, 1 H, Ph-H), 7.35 (s, 1 H, pyrazole-H), 6.85 (d, *J* =3.2 Hz, 1 H, furan-H), 6.52 (dd, *J* =3.3, 1.8 Hz, 1 H, furan-H). ^{13}C-NMR (100 MHz, $CDCl_3$): δ = 162.42, 150.80, 147.64, 145.43, 143.04, 142.08, 139.54, 139.33, 133.49, 132.56, 131.40, 130.78, 127.57, 125.77, 124.93, 123.64, 120.93, 111.57, 108.44, 96.44; HRMS(ESI)[M+H]$^+$ calcd for $C_{20}H_{11}Cl_2F_3N_4O_2$: 467.0289, found : 467.0293.

4-chloro-N-(1-(3-chloro-5-(trifluoromethyl)pyridin-2-yl)-3-(furan-2-yl)-1H-pyrazol-5-yl)benzamide (**8k**). White solid, yield 60.26%, M.p.: 162-164 °C, ^{1}H-NMR (400 MHz, $CDCl_3$) δ11.09 (s, 1 H, NH), 8.66 (d, *J* =1.1 Hz, 1 H, pyridine-H), 8.23 (d, *J* =1.7 Hz, 1 H, pyridine-H), 7.84 – 7.79 (m, 2 H, Ph-H), 7.53 (dd, *J* =1.7, 0.6 Hz, 1 H, furan-H), 7.53 – 7.49 (m, 2 H, Ph-H), 7.29 (s, 1 H, pyrazole-H), 6.84 (dd, *J* =3.4, 0.6 Hz, 1 H, furan-H), 6.52 (dd, *J* =3.4, 1.8 Hz, 1 H, furan-H). ^{13}C-NMR (100 MHz, $CDCl_3$): δ = 162.40, 151.18, 147.54, 145.44, 143.10, 141.77, 139.95, 139.02, 131.73, 129.39, 128.57, 125.53, 124.69, 123.61, 120.90. 111.59, 108.58, 95.82; HRMS(ESI)[M+H]$^+$ calcd for $C_{20}H_{11}Cl_2F_3N_4O_2$: 467.0289, found : 467.0294.

N-(1-(3-chloro-5-(trifluoromethyl)pyridin-2-yl)-3-(furan-2-yl)-1H-pyrazol-5-yl)-4-nitrobenzamide(**8l**). Yellow solid, yield 62.18%, M.p.: 157-159 °C, ^{1}H-NMR (400 MHz, $CDCl_3$) δ11.31 (s, 1 H, NH), 8.68 (s, 1 H, pyridine-H), 8.41 (d, *J* =8.8 Hz, 2 H, Ph-H), 8.26 (d, *J* =1.6 Hz, 1 H, pyridine-H), 8.07 (d, *J* =8.8 Hz, 2 H, Ph-H), 7.55 (d, *J* =1.1 Hz, 1 H, furan-H), 7.35 (s, 1 H, pyrazole-H), 6.88 (d, *J* =3.1 Hz, 1 H, furan-H), 6.53 (dd, *J* =3.3, 1.8 Hz, 1 H, furan-H). ^{13}C-NMR (100 MHz, $CDCl_3$): δ = 168.30, 155.94, 154.72, 152.47, 149.57, 149.36, 148.57, 144.09, 143.95, 143.61, 134.58, 132.87, 131.75, 131.42, 128.98, 117.00, 113.08, 101.80; HRMS(ESI)[M+H]$^+$ calcd for $C_{20}H_{11}ClF_3N_5O_4$: 478.0530, found : 478.0523.

N-(1-(3-chloro-5-(trifluoromethyl)pyridin-2-yl)-3-(furan-2-yl)-1H-pyrazol-5-yl)-4-methoxybenzamide (**8m**). White solid, yield 56.09%, M.p.: 140-142 °C, ^{1}H-NMR (400 MHz, $CDCl_3$) δ10.96 (s, 1 H, NH), 8.69 (s, 1 H, pyridine-H), 8.23 (s, 1 H, pyridine-H), 7.84 (d, *J* =8.8 Hz, 2 H, Ph-H), 7.53 (s, 1 H, furan-H), 7.29 (s, 1 H, pyrazole-H), 7.01 (d, *J* =8.8 Hz, 2 H, Ph-H), 6.84 (d, *J* =3.1 Hz, 1 H, furan-H), 6.54 – 6.49 (m, 1 H, furan-H), 3.89 (s, 3 H, OCH_3). ^{13}C-NMR (100 MHz, $CDCl_3$): δ = 163.05, 162.94, 151.22, 147.67, 145.40, 142.97, 141.79, 140.38, 139.74, 129.07, 125.42, 124.70, 123.63, 120.92, 114.23, 111.50, 108.42, 95.46, 55.51; HRMS(ESI)[M+H]$^+$ calcd for $C_{21}H_{14}ClF_3N_4O_3$: 463.0785, found : 463.0785.

N-(1-(3-chloro-5-(trifluoromethyl)pyridin-2-yl)-3-(furan-2-yl)-1H-pyrazol-5-yl)-6-(trifluoromethyl)picolinamide (**8n**). Yellow solid, yield 82.36%, M.p.: 197-199 °C, ^{1}H-NMR (400 MHz, $CDCl_3$) δ12.29 (s, 1 H, NH), 8.96 (s, 1 H, pyridine-H), 8.79 (s, 1 H, pyridine-H), 8.44 (d, *J*

=8.1 Hz, 1 H, pyridine-H), 8.23 (s, 1 H, pyridine-H), 8.19 (d, *J* =7.9 Hz, 1 H, pyridine-H), 7.54 (s, 1 H, furan-H), 7.35 (s, 1 H, pyrazole-H), 6.86 (d, *J* =3.2 Hz, 1 H, furan-H), 6.53 (d, *J* =1.3 Hz, 1 H, furan-H). ^{13}C-NMR (100 MHz, $CDCl_3$): δ = 159.84, 151.70, 150.79, 147.68, 145.81, 145.45, 143.05, 142.36, 139.55, 139.28, 135.24, 129.69, 129.35, 125.86, 125.52, 124.88, 122.77, 111.58, 108.36, 95.75; HRMS(ESI)$[M+H]^+$ calcd for $C_{20}H_{10}ClF_6N_5O_2$: 502.0505, found : 502.0500.
